# Understanding Men’s Engagement and Disengagement When Seeking Support for Mental Health

**DOI:** 10.1177/15579883231157971

**Published:** 2023-03-07

**Authors:** Minjoo Kwon, Sharon Lawn, Christine Kaine

**Affiliations:** 1 College of Medicine and Public Health, Flinders University, Bedford Park, South Australia, Australia; 2 Lived Experience Australia, Adelaide, South Australia, Australia

**Keywords:** men’s health, mental health, engagement, disengagement, access to care

## Abstract

Men are less likely to utilize health care services compared with women. When it comes to mental health, men have been reported to hold more reluctant attitudes toward engaging with mental health services. Current studies have predominantly been quantitative and focused on understanding effective strategies to promote men’s engagement and why men may avoid help-seeking or may not seek help early; few studies exist of men’s disengagement from services. Much of this research has been undertaken from the services’ perspective. The study reported here attempts to gain better insight into the reasons men give for their disengagement from mental health services and what men say will reengage them back into the system. This research was a secondary analysis of data collected by a national survey conducted by Lived Experience Australia (LEA). Responses of 73 male consumers were gathered and analyzed. Analysis of the responses was split into two themes with associated subthemes: (1) *Why men disengage*: (1.1) Autonomy; (1.2) Professionalism; (1.3) Authenticity; and (1.4) Systemic Barriers; and (2) *What will help men reengage*: (2.1) Clinician-driven reconciliation, (2.2) Community and Peer Workers; and (2.3) Ease of reentry. Findings highlight strategies to prevent disengagement such as creating open and honest therapeutic environments and improving men’s mental health literacy while providing care. Evidence-based approaches to reengage male consumers are suggested along with an emphasis on men’s strong preferences for community-based mental health services and peer workers.

## Introduction

Research from the past two decades has substantiated that men are less likely to utilize health care services compared with women (Affleck et al., 2018; Barrett et al., 2008; Gough & Novikova, 2020; John et al., 2020; Rice et al., 2020; Sagar-Ouriaghli et al., 2019; Seidler, Wilson, Walton et al., 2021). When men do seek services, it is usually motivated by a physical complication or concern, and the time spent in medical consults is much shorter compared with women (Beel et al., 2018; Gough & Novikova, 2020; Smith et al., 2006). Such phenomena are worsened in the context of mental health. Despite existing literatures reporting at least doubled to quadrupled suicide rate in men compared with women, men have been reported to hold more reluctance and negative attitudes toward engaging with mental health services, even at the point of crisis (Levan & Wimer, 2014; Sagar-Ouriaghli et al., 2019). Existing studies have identified traditional masculinity, masculine socialization, and stigma associated with mental health as the major factors leading to adverse mental health outcomes in men (Affleck et al., 2018; Beel et al., 2018; Smith et al., 2006).

### Traditional Masculinity

Traditional masculinity has been associated with characteristics such as being strong, independent, invulnerable, and stoic (Lefkowich et al., 2017; McKenzie et al., 2018; Smith et al., 2006). Societal expectations of what a man should be have led to men’s core identities being founded on these traditional masculine values, ultimately making men reluctant to seek support or help when needed which could be perceived as a threat to their masculine socialization (Beel et al., 2018; Gough & Novikova, 2020; Rice et al., 2020). Staigar et al.’s (2020) interviews with men who experience depression reported that when men displayed their struggle in social or workplace settings, they experienced criticism and name-calling (i.e., lazy, incapable) rather than support. Hence, these traditional values and social stigma along with men’s personal experiences became a barrier for men to even acknowledge their struggles in the first place.

Consequently, men may choose to neglect their mental health and refrain from discussing topics of mental health and self-care with others, contributing to low mental health literacy among men and further delayed help-seeking (John et al., 2020; Sagar-Ouriaghli et al., 2019; Seidler et al., 2020; Seidler, Wilson, Walton et al., 2021). High prevalence of risk-taking behaviors such as alcohol and substance abuse in men was also interpreted as an outcome of men’s reduced ability to recognize their mental health decline, tendency to socially withdraw during difficult life events, and preference for tangible mechanical coping mechanisms rather than having to be emotionally vulnerable (Gough & Novikova, 2020; McKenzie et al., 2018; Smith et al., 2006). Thus, men tend to address their mental health issues either when it has manifested physically or when prompted by trusted others such as family and close friends, and access mental health services and support indirectly (McKenzie et al., 2018; Seidler, Wilson, Walton et al., 2021).

### Gender-Sensitive Strategies to Help Men’s Engagement

With the understanding of traditional masculinity, suggested strategies to improve men’s health services utilization can be sorted into two main approaches: reframing masculinity and community engagement. Reframing masculinity encourages change of perspective toward the traditional masculine values men already have rather than defying them (Seidler et al., 2020). For example, McGraw et al. (2021) and Sagar-Ouriaghli et al. (2019) emphasize that not all masculine qualities are detrimental but some such as being competitive and wanting to succeed can be used to help men persevere and take up the responsibility in managing their health. In fact, participants of Staigar et al.’s (2020) qualitative research viewed their mental health challenge as a “wake-up call” or an opportunity to change themselves. Along with encouraging positive values, problem-solving and activity-based treatments were recommended to give men an active role in their health management, thus helping men to feel more independent and in control of their situation (Gough & Novikova, 2020; Seidler, Wilson, Walton et al., 2021).

The most significant benefit of community-based approaches for men has been reducing sociocultural stigmatization (Rice et al., 2020; Seidler, Wilson, Kealy et al., 2021; Staigar et al., 2020). Being able to interact with other men in activity-oriented community groups (such as sporting venues or other nonclinical community locations where men may congregate in their daily life) helped men to change their self-perception on mental health management, and such settings allowed men to find rolemodels within the community, which motivated them to continue taking care of themselves (Lefkowich et al., 2017; Sagar-Ouriaghli et al., 2019). Being part of community enhanced a sense of social connection that can be a “buffer” in times of crisis as well as improving adherence by giving men a sense of responsibility and partnership via delegation (McKenzie et al., 2018).

### Beyond Traditional Masculinity

While addressing the traditional model, more recent studies have acknowledged the need to view masculinity as a multidimensional construct and appreciate more contemporary models of masculinity (McGraw et al., 2021; McKenzie et al., 2018; Staigar et al., 2020). Despite traditional masculinity being able to provide explanations for men’s help-seeking patterns, not all men have the same perception of masculinity. They have diverse personal experiences; thus, more personalized approaches are likely required for each individual (Kealy et al., 2021; Sagar-Ouriaghli et al., 2019).

### Our Research

The majority of male-specific literatures available address initial help-seeking patterns of men through the lens of traditional masculinity (Affleck et al., 2018; Gough & Novikova, 2020; Lefkowich et al., 2017; McKenzie et al., 2018; Sagar-Ouriaghli et al., 2019; Smith et al., 2006; Staigar et al., 2020). However, there are limited male-specific studies beyond this point on what happens once men overcome the initial barriers and engage with the services, what makes some men disengage from mental health services, and what could be done to support their reengagement. There are non-gender-specific papers and literature reviews addressing the issue of “dropout,” which highlight the importance of therapeutic alliance, systematic approaches, and patient-centered holistic care (Barrett et al., 2008; Chen et al., 2017; Cooper & Colkins, 2015; Henzen et al., 2016). A study by Kealy et al. (2021) explored men’s preference in types of psychotherapy and has emphasized the need for patients’ preferences to be acknowledged by clinicians. One study specifically focused on men’s dropout from mental health services; however, it mainly provided quantitative data (Seidler, Wilson, Kealy et al., 2021). To our knowledge, Seidler et al.’s (2018) was the only qualitative study exploring factors affecting treatment engagement in men diagnosed with depression, which highlighted the importance of patient-centered, structured, transparent, and gender-sensitive approach.

This article aims to bridge the gap between what is understood of men’s engagement with mental health services in existing literatures and what the actual consumers experience while attempting to gain better insight into the reasons men give for their disengagement from various mental health services and what men say will reengage them back into this system of mental health care. Our research also aims to provide in-depth qualitative data from male consumers who have had recent contact with mental health services, which can be used to further inform implementation of existing suggested strategies, validity of masculine socialization models, and consumer-focused studies.

## Method

### Ethical Statement

This project, and the original larger study from which data for men was drawn, was approved by Flinders University Human Research Ethics Committee (No. 5129).

The study involved a secondary analysis of data collected by a national survey (Kaine & Lawn, 2021) conducted by Lived Experience Australia (LEA), which is an Australian national mental health consumer and carer advocacy organization (https://www.livedexperienceaustralia.com.au/). The objective of the survey was to gain a better understanding of Australian mental health consumers’ and family carers’ experiences of engagement and disengagement with mental health care services.

The anonymous survey was sent out electronically via SurveyMonkey to LEA’s email list of more than 2,000 “friends,” with the survey link also distributed voluntarily by other collaborative mental health consumer and carer advocacy peaks and organizations at state and national level. The survey was open for 3 weeks and 535 individuals commenced the survey (404 identified as consumers and 131 identified as family carers). Participants could elect not to answer questions and their consent to participant was provided electronically via the online site through their commencement of the survey. While 404 consumers commenced the survey, with a mean average completion rate of 99% for the eight upfront demographic questions, fewer (*n* = 285) commenced questions in the main section of the survey and went on to answer the 23 questions in that section, with a mean average completion rate of 84.3% (range = 63.2–100%). Within this larger sample, there were a total of 73 male consumers who participated in the survey.

The survey contained 42 questions applicable for consumers, consisting of both quantitative and qualitative questions (see Box 1). In all questions in the main section of the survey, participants could provide detailed qualitative comments to expand on their responses. Responses to Questions 35 to 42 were excluded from this analysis as the nature of the questions was more focused on accessibility to private mental health services. This article focuses on report of men’s qualitative responses.

**Box 1.** Outline of Survey Questions (Refer to the Appendix for Full Survey Questions)

Preliminary section • (Q1–Q8) Eight questions seeking demographic information (e.g., age, gender, location) Main section • (Q9–10, 14–29) 19 questions asking about: ○ Type of services accessed in the last 5 years ○ Likert-rated or yes/no responses to questions asking about services used and why, access to services, perceived quality of services and health professionals, disengagement and why, and reengagement and with whom. ○ Each of these questions provided the opportunity for participants to make further qualitative comments. • (Q11–13) Three questions asking about the use of digital mental health services • (Q30–34) Five qualitative questions about: ○ What would help people to stay engaged or reengage with services ○ Perceptions about what happens to people after disengagement ○ Preferences for services that are currently inaccessible

### Qualitative Analysis

Participants provided detailed qualitative responses. Initially, all survey responses across all survey questions were read iteratively. During the process, tentative themes were formulated and discussed by the researchers, and reoccurring issues and comments were noted down on a Word document (Document 1). Then, the survey responses were read again, and all meaningful and relevant quotes were added to Document 1 in a refined or shorter form that highlighted the main points in each tentative theme area. Document 1 was then read repeatedly, and relevant comments and key points were categorized and then grouped into general categories according to relevance, enabling further robust discussion by the researchers (Document 2). A total of 11 general categories were formed. Before finalization of the theme structure and analysis, raw survey responses and Documents 1 and 2 were revisited to ensure all relevant comments were included and also to evaluate the legitimacy and parsimony of the final themes and subthemes formulated. In the end, consideration of the underpinning research question produced two main themes and seven subthemes.

During this qualitative analysis process, tentative themes were formulated and discussed by the first two researchers (MK, SL) via regularly meeting held every 2 to 4 weeks to discuss, debate, and agree on the themes and categorization. These meetings involved discussing survey responses, and sharing analyzed documents, lists of keywords, and word diagrams. This process resulted in a list of keywords and topics that were then grouped into general categories according to relevance, enabling further robust discussion by all three members of the researcher team.

This analysis method was developed based on Seidelr’s (1998)“Noticing, Collecting and Thinking” qualitative data analysis process, as described in Box 2. Please refer to Supplementary Document for detailed process summary.

**Box 2.**
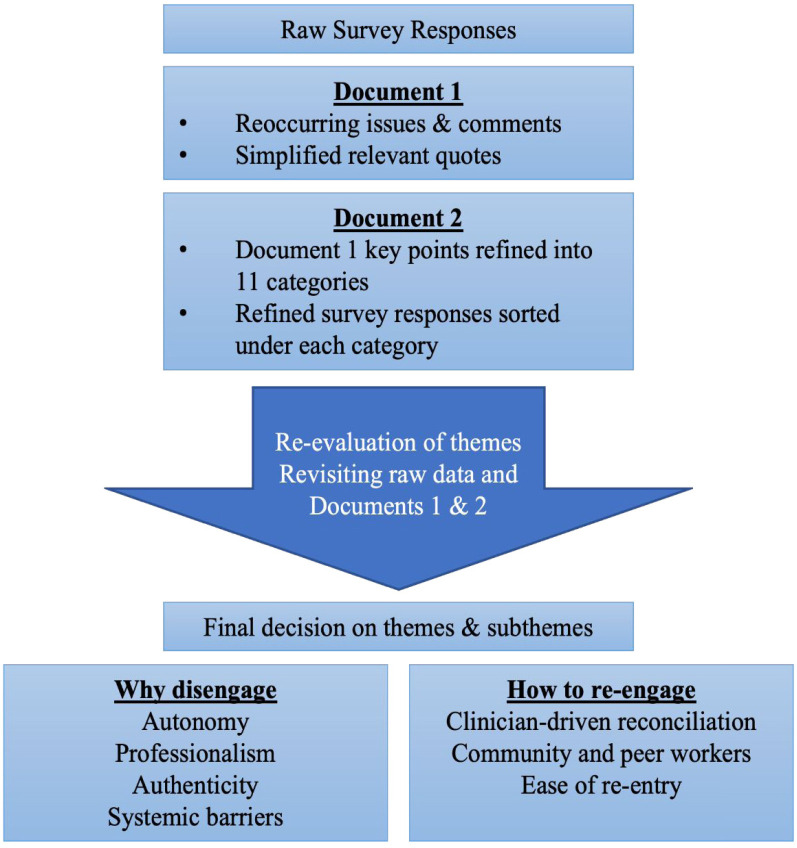
Qualitative Analysis Flowchart

## Results

A total of 76 men participated in this research. Analysis of qualitative data from the survey produced two main themes, each comprising a series of subthemes:

(1) *Why men disengage*: (1.1) Autonomy; (1.2) Professionalism; (1.3) Authenticity; and (1.4) Systemic Barriers
(2) *What will help men reengage*: (2.1) Clinician-driven reconciliation, (2.2) Community and Peer Workers; and (2.3) Ease of reentry

### Why Men Disengage

#### Autonomy

Men’s comments were underpinned by a strong desire for autonomy and control in decision-making and interactions with health professionals. Survey responses identified that failure to respect the participant’s autonomy in clinical practice contributed to disengagement. Participants often expressed that they did not have “a choice” during their care, often due to absences of transparency and having limited options for treatments. It was further commented that health professionals failed to integrate the patient’s perspective or seek any patient input. These circumstances ultimately led participants to feel that they had little to no control in what would happen to them as recipients of services, as if the clinicians’ ideas were being forced onto them. In addition, having their autonomy disregarded in clinician–patient interactions made some participants feel stereotyped, as they thought the health professionals made assumptions about them that were unfounded:

Psychiatrist and social workers went by my ‘notes’ and seemed to have made up their minds without any input from me. (Q17, Participant 10)

(I need) services that listen and respond to my needs and experiences, not have their own ideas about what is best for me . . . I know what my needs are. . . (Q19, Participant 1)

The strong desire for autonomy appeared to have led some participants to self-evaluate their recovery without discussing their circumstances with health professionals, leading to premature disengagement. The participants explained that they did not realize the effectiveness of treatment at the time and stopped getting the support:

. . . I used to have the attitude of “I’m all better now” scenario and stop seeing my supports and stop taking my medication. (Q21, Participant 27)

#### Professionalism and Competency

Participants commonly complained about the lack of professionalism among mental health service providers and that this disappointment caused them to cease therapeutic relationships. Such consensus demonstrated that the participants placed a certain level of expectation on health care professionals and were often disappointed when those standards were not met. Some participants expressed that the health professionals lacked versatility in types of treatments they could offer and yet failed to be transparent with their clinical limitations. Due to this, participants did not have a clear sense upfront of the structure of treatments, leading them to perceive that they were not receiving the correct care needed, resulting in delayed treatment or received no referral to services that could have helped them. This appears to have contributed to participants feeling that they were forced into inappropriate treatments and ultimately losing trust in their health care provider. The importance of health professionals’ ability to provide a range of care was also evident in participant comments:

I ended it because people were no longer responding to my needs or listening to me, rather they had their own idea of what was best for me. This did not align with what I actually needed and felt that I was wasting their time and they were wasting mine. (Q19, Participant 1)

Several participants commented that their health professional retired, moved, or decided to cease seeing their patients without communicating or referring them to alternative services. This discouraged those participants who then gave up seeking other support and disengaged, subsequently “slipping through the gap.”

Some participants shared that their health professional “dropped their guard” and lost professional attitude. In one case (as exemplified by Participant 1’s comment to Q23), the participant decided to discontinue the treatment despite having communicated their concerns to the health professional and receiving their apology. Other subprofessional behaviors witnessed by the participants included health professionals appearing confused or unsure about the treatment they were providing and having no consensus between health care providers. These circumstances all contributed to a breakdown in trust in the health professionals and their treatments:

I felt that the clinician was unsure what to do. He was tired, as he recently had his first child and I think his guard dropped somewhat. I’ve spoken to him since and discussed this with him. He was very apologetic and (said he) didn’t realise, but I felt frustrated. . . so thought let’s just do this on my own and so I did. (Q23, Participant 1)

No level of consistency. . . being told something by one staff and then later told the opposite by other staff. . . I was left confused. (Q24, Participant 3)

#### Authenticity

Almost all participants expressed views indicating that they perceived health care providers were not genuinely interested in them as a person and not genuinely empathic toward their individual situation. Perceived lack of listening and effort for rapport building by health professionals led to many participants questioning the authenticity of the care being provided:

Some participants expressed that they needed more sensitive care and support, and more effort on the part of health professionals to remove stigma and judgment while the care is being provided:

. . . (they were) judgemental, inconsiderate, out of touch, invalidating, bossy, overconfident, strange personal theories about treatments and what works, blaming me for not being well yet, patronising, indifferent, asks bizarre questions. (Q17, Participant 6)

Patronising, thinking I am lesser than them, not disclosing anything yet expecting us to disclose traumatic events. This is far too common with psychiatrists. (Q17, Participant 13)

#### Systemic Barriers

Three main issues within the Australian health care system have been identified by the participants as barriers preventing men’s engagement in mental health support: consistency, aftercare, and accessibility. In line with the larger study, many male respondents complained about a lack of consistency and coordination while receiving care. While visiting the same service, they reported often being seen by different clinicians although this was not their preference. They also reported a lack of coordination within the clinic and between clinicians that many perceived as adding further burden to their attempts to receive appropriate care.

Participants described a similar pattern of disengagement caused by services’ failure to provide appropriate aftercare. Several participants shared that they received no referral to any related services or follow-up support after visiting health care providers during crisis. Several participants also expressed that there was no effort from the services to continue communication or reach out first, eventually leading them to “slip through the gap” in care and be lost to follow-up.

Problems with access to mental health services were a common reason participants gave for why they refrained from receiving mental health care. Cost of the services and the limited number of financially covered appointments running out also limited their access to services:

I have engaged with dozens of providers over a decade. . . Navigating the mental health system is an arduous grind, trying to figure out what’s wrong, what you need and what helps. There is a massive amount of uncertainty here. . . Not only that but cost is an enormous factor. . . Therapy is helpful but can take several years to see results. (Q21, Participant 16)

### What Can Help Men Reengage

#### Clinician-Initiated Feedback and Reconciliation

Many participants who said that they had disengaged from support expressed that they had wished for an opportunity to provide feedback to health professionals and the service and have an open discussion to mend the therapeutic relationship. One participant suggested that an online, third-party platform for feedback would help them to provide honest feedback. Several participants described their desire for transparent and honest communication with health professionals, and that acknowledging the problems was an important part of mending, as well as health professionals being open to receiving constructive criticism. It was also apparent that participants preferred such processes to be initiated by the health services or health professionals:

Actually talk to the consumer. . . to get honest feedback about the service, definitely best done by impartial third party because there is a large power imbalance. . . Foster an environment of open dialogue that allows the patient the space, if required, to express neediness or dissatisfaction, even if it may not be realistic. Sometimes the patient needs to vent to discover their expectations or conclusions are unrealistic. (Q31, Participant 12)

#### Community and Peer Workers

Throughout the survey, participants shared many positive experiences and views on involving peer workers in their treatment. Even those participants who had not had peer workers involved in their past care expressed strong willingness to try accessing the service again via peer workers. Participants generally perceived that support from someone with a “true lived experience” who could empathize with them would help them to feel better understood, and some participants expressed that their contact with peer workers motivated them to stay engaged in services:

The best thing for me was to have regular visits from a social worker who had lived experience. They understood how difficult getting help could be and acted as a dependable rock I could rely on for support. They helped me to co-ordinate the extra supports I required, and to liaise between the deterrent specialists. Patients who have disengaged need an empathetic, understanding SINGLE point of contact, to ensure continuity of support. (Q33, Participant 12)

A community-based approach for support was also highly preferred. Participants’ responses substantiated the need for lived experience support groups and similar community activity groups. Many participants described how focusing on human and social factors helped them to feel connected and find more meaning during the care. Furthermore, some participants expressed that they would like to get recommendations from peer workers to better manage their mental health. More accessible community services, such as 24/7 phone lines and local free mental health services facilitated by peer workers, were also mentioned by some participants as beneficial to supporting them to reengage with services:

Sustained therapy is challenging. When I found some peers to connect with, that’s what made it possible for me to stay engaged. (Q21, Participant 9)

#### Ease of Reentry

Participants expressed the need for an easier, more transparent pathway and structure for reentry into mental health care. First, participants complained about long wait times for those individuals who did not have access to private services. It appeared that those who wanted to reengage with their previous provider were often discouraged by longer wait times and were reluctant to try other services due to having to repeat their stories and the assessment process. Participants demonstrated a strong preference for a clear and consistent point of contact and, if this was unavailable, then better coordination in the health care system that could help them to reenter without worrying about having to repeat the entire process:

(We need) Better integrate services together, keep records in one place, have a case manager for complex cases to co-ordinate between GP, psychologist, psychiatrist, Centrelink, hospitals. Stop having to reprove illness to each party. (Q30, Participant 14)

As a similar solution, an active post-care approach from the health service was also suggested. Participants wanted to be contacted by their services after disengaging, for a chance to share their feedback or as a general follow-up. By keeping them in a loop of communication even after disengaging, it was suggested that this could potentially encourage reengagement with the service and create a more welcoming environment. In addition, community-based outreach programs were also favored along with better education on how to navigate the health system and campaigns to encourage engagement:

I think continual communication (can help people stay engaged) . . . either via a mobile phone of letter . . . requesting some form of response to ascertain that they are still okay and that the service would be always made available if need be . . . keeping them in the loop of communication making sure they are okay. . . and welcoming them back should they require the service and how they are to do that first point of call. (Q30, Participant 12)

Accessibility improvement was also required at a systemic level. This included access to a greater variety of community-based services and 24/7 services, offering more choice and control, other than the hospital emergency department. Lower costs for services and a higher number of medical visits covered by Medicare were also suggested. Some participants expressed frustration regarding the need for re-referral and how it lengthens the wait time to see a mental health professional and expressed that not having to renew referrals would ease their reentry into the health care system.

## Discussion

### Addressing Traditional Masculinity

Data analysis identified concurrent themes in men’s disengagement with traditional masculinity. Most prominently, the traditional value of independence appears to have manifested as a strong desire for autonomy and a sense of control in their mental health care (Seidler et al., 2018; Seidler, Wilson, Walton et al., 2021). Seeking patient input and perspective, having a variety of options and being able to make decisions on their treatments were main factors influencing the level of sense of autonomy men experience in clinical settings, findings that mirror previous research with men (Seidler et al., 2018).

In some instances, independence combined with the issue of poor mental health literacy led to disengagement regardless of the level of autonomy experienced during care by the participant (John et al., 2020; Sagar-Ouriaghli et al., 2019). Some participants either were or witnessed cases where men made self-evaluation of progress without consulting with mental health professionals and prematurely disengaged from support believing they were cured and do not need further help. Therefore, it would be important for clinicians to incorporate mental health education from early therapeutic interaction to help men understand the role of mental health care in their recovery and what might happen if they disengage without proper aftercare planning. This can also help men gain more sense of control over their condition that could help them to have better insight, be better at identifying early signs and symptoms and become proactive. With improved mental health literacy, they will be able to evaluate their mental health status more accurately even after disengagement, which will enable their timely return to mental health services without having to wait until the point of crisis.

Interestingly, there were almost no comments on social stigma being a cause of disengagement or barrier to reengagement. It may be possible to interpret that the participants overcome the fear of social stigma when they first contact the mental health care services and continue to be indifferent to the stigma. There were some reports of disengagements caused by clinicians’ stereotypes during treatment (discussed in *1.1 Autonomy*), but this appeared to be more associated with the mental health conditions itself rather than masculinity.

### Expectation on Clinicians

As discussed in *(1.2) Professionalism and competency* and *(2.1) Clinician-initiated feedback and reconciliation*, the influence of the mental health service providers was significant in men’s disengagement and reengagement. Participants expressed difficulty developing trust and meaningful therapeutic relationships when the health care providers could not maintain a good level of professionalism and competency. The results reported that even if the health professionals are unable to offer various ranges of care, men appreciate the honesty of clinicians if they identify their clinical limitations and put in a genuine effort to support the male consumers. It appeared that while clinical skills are important factors that help men to build trust toward their care provider, being transparent with treatment, setting clear goals or agendas, and demonstrating effective communication skills are also important in helping men to feel that they are working in a partnership rather than being forced or coerced into treatments, thus having more sense of control over their recovery journey.

The concept of epistemic trust is worth consideration in relation to the findings here. Epistemic trust has been described as an individual’s judgment that the information provided by their health professional is relevant, significant, credible, and worth incorporating into their life (Campbell et al., 2021; Fonagy et al., 2015). It is a specific form of trust in the knowledge delivered that also involves transparency in the health care provider/patient relationship, and which may be a central requirement for treatment effectiveness and for avoiding disengagement. It appears to be of particular importance to men in their decisions to engage, disengage, and reengage with mental health services. As already suggested in existing literature, health professionals actively addressing safety and power dynamics earlier on in health management encounters could be a strategy to start discussing a healthy and trusting clinical environment in their interactions with male patients (Barrett et al., 2008; Beel et al., 2018; Chen et al., 2017; Martínez-Martínez et al., 2021; Seidler et al., 2018).

To reengage male patients, mental health care providers should be actively encouraged to reach out to those who have disengaged. It was unclear whether the participants wanted to just provide feedback and be heard or if they were willing to reengage with the same health care provider; however, such a feedback process could aid health professionals to evaluate the quality of care they currently provide, reflect on the feedback they receive, and use the information to improve their clinical approach.

### Community Involvement and Peer Workers

There was a strong preference for community-based care (available in the person’s everyday community, beyond the more clinically focused mental health services) and peer worker services (delivered by individuals with their own lived experience of mental health conditions who are now in recovery) among the participants, which is supportive of the existing consensus endorsing community-based strategies to engage men (Gough & Novikova, 2020; Hans & Hiller, 2013; McKenzie et al., 2018; Sagar-Ouriaghli et al., 2019; Staigar et al., 2020). Of those participants who have utilized community-based services or peer workers, the authenticity of people providing care appeared to have attracted them. Having a care provider with a lived-experience appeared to have allowed participants to feel deeply understood, leading to more meaningful therapeutic relationship.

In some cases, peer workers appeared to have addressed the coordination issue within the healthcare system by managing liaison between health disciplines. Perhaps a more active involvement of peer workers or having case workers assigned to clients who may need to coordinate multidisciplinary services could prevent clients from becoming fatigued through the process. It would be beneficial for mental health professionals to make an early review of the person’s situation holistically, to make timely referrals to these services, and therefore help the person to stay engaged.

In summary, community-based approaches and peer workers could prevent disengagement. It may even be a preferred reengagement pathway for those who have been let down by private services. From an aftercare perspective, community services could allow a spontaneous exchange of information regarding mental health and continue to improve men’s mental health literacy. Peers also may be able to screen the person’s current status and encourage reengagement before adverse events.

### Australian Health care System

Systemic barriers preventing the reentry of men into the mental health care system have been identified. The most prominent issue was the long wait time caused by systemic factors such as the requirement for a referral, long waitlist, and general accessibility. Participants appeared to be prone to lose motivation to continue seeking help when the wait time increased. The desire for a greater number of services covered by mental health care plans to reduce the need for renewal and more low-cost subsidized services was apparent. This potentially could be why participants displayed strong preferences for general practitioners when seeking mental health support, as the wait times tend to be shorter, and the participants often already have established therapeutic relationships.

Furthermore, participants expressed their reluctance to repeat the process of help-seeking, such as having to repeat their stories, finding clinicians who satisfy personal preferences, and having to rebuild the therapeutic relationship from the ground. It was suggested there should be a central database for the patient record, such that if the patient needs to see another mental health care provider, health professionals can access the patient information without the patient having to explain themselves all over again. This may also address the issue of poor coordination within the health system and ensure consistent multidisciplinary support for the person.

There also was a demand for more 24/7 after-hours services. A few participants expressed that they do not particularly require longitudinal care but instead need a safe space devoted to people in a mental health crisis with peer support available, and after-hours consults available for those who cannot attend appointments during the typical 9:00 a.m. to 5:00 p.m. business hours.

Australian mental health system reforms were introduced from 2020 to enhance alternatives to hospital emergency departments as the primary mode of help-seeking for mental health crisis, with the establishment of a series of Adult Mental Health Centres designed to meet some of the needs described above (Australian Government Department of Health and Aged Care, 2021). However, it is yet unclear how men are engaging with this new addition to the community mental health system in Australia.

## Clinical Implication

It is recommended that mental health care professionals set clear agendas and goals at an early stage of the therapeutic relationship when interacting with male consumers (Chen et al., 2017). It is integral to maintain transparency during the consult and offer men a reasonable range of services they can access. This could be achieved by setting review points in the first consultation between the health professional and the consumer to openly discuss any concerns about the treatments they are receiving. It would be crucial for the health professional to create a safe clinical environment and give the consumer regular reassurance that their views are respected.

Health professionals also should integrate mental health education into their practice to help male consumers gain a better understanding of their conditions and to allow them to make a timely return to the service if their mental health starts deteriorating after disengagement. Aftercare such as follow-up calls and messages should be provided to those who decide to discontinue a therapeutic relationship either to remind them that support is available when needed or to prevent men from falling through the gaps.

It could be helpful for both healthcare providers and consumers if a more transparent feedback system for service users to voice their concerns were established by services. This could allow those who had disengaged to feel safe and encouraged to return to services and allow health professionals to provide better personalized care.

At a systemic level, strategies to simplify reentry pathways would help men to reengage. A system to ensure coordination between disciplines and services can also benefit men to build more trust in the system and be less concerned about processes for reengagement.

## Limitations

This research had a number of limitations. The sample of men completing the survey was relatively small and the short length of survey time (3 weeks) also limited the sample. Specific ethnic, cultural, and sexual minority populations were also underrepresented, and their reasons for engagement and disengagement may vary. Reliability testing of the survey was not undertaken, and participants could opt out of answering some questions. The type of mental health conditions or diagnosis of individual participants was not a part of the survey. The sample recruited may not reflect the broader community of people with mental health issues, and findings of this Australian survey may not be generalizable to the experience of men, more broadly, or to other countries. In addition, the views of younger males (18–24 years) could not be distinguished due to how this question was asked in the survey, and people below 18 years were excluded. These limitations are important, given that mental health conditions often begin during childhood, adolescence, and early adulthood.

## Conclusion

This study corroborates strategies suggested by existing research on how to encourage men’s engagement in mental health care and the need for a gender-sensitive approach considering traditional masculinity and the significance of community involvement (Gough & Novikova, 2020; Lefkowich et al., 2017; McKenzie et al., 2018; Sagar-Ouriaghli et al., 2019; Seidler et al., 2018; Staigar et al., 2020). The findings also emphasize the health professional’s ability to significantly influence men’s engagement with mental health care. Ensuring sense of autonomy and self-determination are important underpinning principles for practice with all individuals seeking and receiving support for their mental health concerns given issues of stigma and marginalization. This study has confirmed that these principles are particularly important for men, and their absence can have a significant influence on men’s decisions to engage, disengage, and reengage with mental health services. Future research could investigate whether suggested feedback systems for mental health care providers and disengaged male consumers are effective in encouraging reengagement. Research focused on disengagement patterns of younger male consumers is also needed.

## Supplemental Material

sj-docx-1-jmh-10.1177_15579883231157971 – Supplemental material for Understanding Men’s Engagement and Disengagement When Seeking Support for Mental Health Click here for additional data file. Supplemental material, sj-docx-1-jmh-10.1177_15579883231157971 for Understanding Men’s Engagement and Disengagement When Seeking Support for Mental Health by Minjoo Kwon, Sharon Lawn and Christine Kaine in American Journal of Men's Health

## Appendix

### Survey Questions

**Q1. In which state/territory do you live?**

Victoria

South Australia

New South Wales

Queensland

Tasmania

Western Australia

Northern Territory

Australian Capital Territory

**Q2. Are you located in a:**

Capital city

Regional center

Remote town

**Q3. Are you**

Male, Female, other

**Q4. What is your age range?**

Under 20 years

20–39 years

40–49 years

50–59 years

60–69 years

70–79 years

80 years or above

**Q5. Are you of Aboriginal or Torres Strait Islander descent?**

Yes

No

Prefer not to answer

**Q6. What is your country of birth if not Australia?**

**Q7. What language do you mostly speak at home?**

English

Other (please specify below)

**Q8. Are you completing this survey as a:**

Consumer (someone with a mental illness or experience of mental ill-health)

Carer or family member

**CONSUMER ONLY QUESTIONS**

**Q9. Select from the following options the one which best describes what services, health professional or supports you have mainly used in the past 5 years for your mental health:**

Public mental health services/hospitals/community teams

Private mental health services/hospitals

My GP

Only used a Private Psychiatrist

A Psychologist, counselor/therapist

Veteran supports

Peer support (organized or unorganized)

Telehealth

Online or digital resources or Apps

Other (please specify)

**Q10. Please explain the main reasons why you use this as your primary source of mental health support? Rate each of the following reasons:**

Answer Choices

a. Major Contributing Reason

b. Contributing Reason

c. Not a Contributing Reason

d. Not Applicable

I don’t have to wait too long to see someone

The service meets my needs

They don’t make me repeat my story too much

They listen to me

They include/collaborate with me

I feel I have some say or control in making decisions

They include my family/carer

They respect my privacy if I don’t want to include my family

I trust them

I feel safe here

I don’t feel judged/stigmatized by them

I can afford to pay for this service

Limited options/choice of service providers in my area

I have a consistent worker

They are organized and coordinate the support services I need

They seem to have a clear plan/goals

I am able to see a worker whose gender is of my choosing

Other (please specify)

**Q11. If you used digital resources or Apps, which of the following influenced your decision to commence an online course for mental health and well-being? (Select all that apply)**

My health professional recommended that I do the course

My friends or family recommended that I do the course

It was convenient for me to access due to limited availability of other mental health services in my local area

It was convenient for me to access due to my limited availability to attend a face-to-face treatment

It was convenient for me to access outside of the normal consultation (business) hours

The cost of face-to-face services

I chose to remain anonymous and limit personal information shared

I wanted to control the level of contact I have with my service provider (e.g., no contact with doctor, only receive feedback via email)

I was on the wait list for other services

I previously used other services or treatments but was dissatisfied

I previously used or was still using other services but I wanted to try something new

I prefer to use digital services rather than face-to-face services

The reputation of the institutes providing the online course

The scientific evidence supporting the online course

Not applicable

Other (please specify)

**Q12. At the time when you enrolled into an online course, what other support or treatment were you receiving to manage or improve your mental health and well-being? (Select all that apply)**

None

Another online program

Medication

Face-to-face therapy with mental health professional (e.g., psychiatrist, psychologist, social worker, mental health worker)

Group therapy (including as an outpatient in a hospital setting)

Participation in an exercise group subsidized under Mental Health Treatment Plan

Alternative medicine (e.g., naturopathy, homeopathy, acupuncture)

Not Applicable

Other (please specify)

**Q13. If you didn’t complete the online course, please indicate why: (Select all that apply)**

I was not ready to commit to an online course at the time

I wanted to discuss it first with my health professional

I no longer felt that I needed to do the course

The cost of the course was too high

I accessed another service and/or started another treatment

I experienced technical difficulties

I didn’t improve

Not Applicable

Other (please specify)

**Q14. After you realized you needed support, were you able to access a mental health service or a health professional in a reasonable time?**

Yes

No

Please comment

**Q15. Were there particular qualities of the service that helped you to feel more comfortable engaging with them?**

Yes

No

Please comment

**Q16. Were there particular qualities of the health professional that helped you to feel more comfortable engaging with them?**

Yes

No

Please comment

**Q17. Were there particular things about them that made you feel uncomfortable and not want to engage with them?**

Yes

No

Please comment

**Q18. Did this health professional or service help you for the length of time you felt you needed?**

Yes

No

Please comment

**Q19. If no, did you or the health professional or service make the decision to end your support?**

Myself

Service

Other

Unsure

Please comment

**Q20. If no, did/are you intending to find alternative help for your mental health issues?**

Yes

No

**Q21. Do you think that disengagement (stopping) use of mental health services is an issue for a lot of people?**

Yes

No

Unsure

Please explain the main reasons for your response

**Q22. Did the health professional or service give you and your family and carer sufficient notice of your impending discharge?**

Yes

No

Unsure

Please explain the main reasons for your response

**Q23. Thinking about the health professional or service that you decided not to engage with (continue with) in the past, what was the primary reason you decided to stop (disengage)?**

Answer choices

a. Major Contributing Reason

b. Contributing Reason

c. Not a Contributing Reason

d. Unsure

Wait times were too long

A referral was required but I didn’t get one when I asked

Discharged from mental health professional/mental health service with no follow-up

The service didn’t meet my needs (wrong care)

The service didn’t offer me the right type of support that I needed

Cost was prohibitive/I couldn’t afford to pay for it

Limited options/choice of service providers in my area

Worker changed frequently/no consistent worker

Told that I didn’t meet/no longer met criteria of the service

Lack of plan/goals/didn’t seem to be progressing/going anywhere

Didn’t need the full number of appointments as I felt better quickly

Other

Please comment

**Q24. From a personal perspective, thinking about services that you decided not to engage with (continue with) in the past, what was the primary reason you decided to stop (disengage)?**

Answer choices

a. Major Contributing Reason

b. Contributing Reason

c. Not a Contributing Reason

d. Unsure

Made me repeat my story too much

Didn’t listen to me

Didn’t include/collaborate with me

I felt I had little say or control in making decisions

Didn’t include my family/carer

My family was included and I didn’t like that

I didn’t trust them

I didn’t feel safe there

I felt judged / stigmatized by them

I was forgotten about

Decided to stop because another service was better for me

I felt better and had recovered

Decided my family or close friends supported me better

Peer support worker was best suited to my needs

Community support groups were best for me

Other

Please comment:

**Q25. How did you find the communication and collaboration between health professionals and/or services?**

No coordination

There was not a referral to other services

I was discharged from hospital with no referral or follow up

I was discharged from community services before I was ready

I felt I fell through the cracks

It wasn’t clear who I could contact when I needed to

I didn’t; have a consistent person who I could contact or speak to

Each time I contacted them for help, I had to retell my story/they didn’t seem to remember my situation, needs or preferences

**Q26. If you decided not to continue but need support now or likely to in the future, which health professional or service will you try to reengage with? Please tick all that are relevant to you**

None

Psychiatrist

Psychologist

Social worker

Public community mental health

Public mental health inpatient unit

Private psychiatric hospital

Headspace

Counselor/therapist

GP

Peer worker

Other

Please comment

**Q27. If you have found yourself in a crisis, would you please indicate whether any of the following contributed to the deterioration in your condition:**

Couldn’t access support when needed

Didn’t have a regular health professional that I could get help from

Not connected to existing services

Regular health professional not available

Social issues

Other

Please comment

**Q28. When you found yourself in a crisis, did you seek help through an emergency department?**

Yes

No

Not applicable

**Q29. If you answered yes, and you were not admitted to hospital, could you please explain what happened after you were discharged?**

Went home

Went to my family or friends

No referral to mental health services

No referral to a health professional

Given a discharge letter to my GP

Given a Referral to a community mental health service

Organized consultation with community mental health team

Referral to a psychiatrist or psychologist

No follow-up

Other

Please comment

**Q30. What do you think would help people stay engaged with health professionals or services or return to a health professional or service to receive support for their mental health? Please tell us your ideas.**

**Q31. What would assist/support people to reengage with services where they had previously disengaged from them? Please tell us your ideas.**

**Q32. From your experience, what do people do/what happens to them after they disengage with services? Please comment:**

**Q33. How do you think services could best reengage with people who have disengaged with mental health support from services? Please tell us your ideas**

**Q34. What services would you like to access to support your mental health and well-being that you can’t access at the moment? Please comment**
