# Supplementary figures and images for: Understanding Men’s Engagement and Disengagement When Seeking Support for Mental Health
Source: Am J Mens Health. 2023 Mar 7;17(2):15579883231157971. doi: 10.1177/15579883231157971 (PMC9996733; doi:10.1177/15579883231157971)

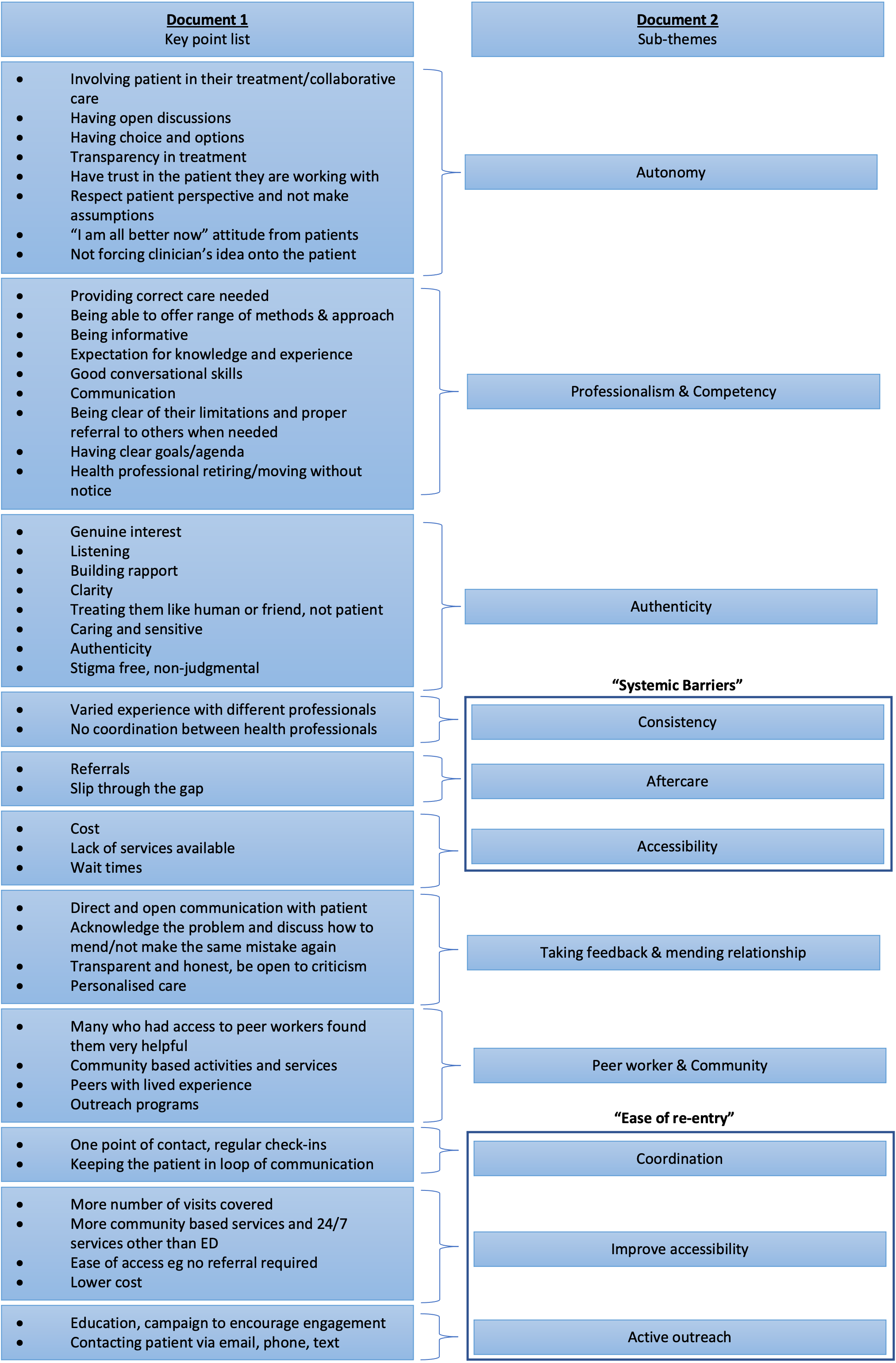


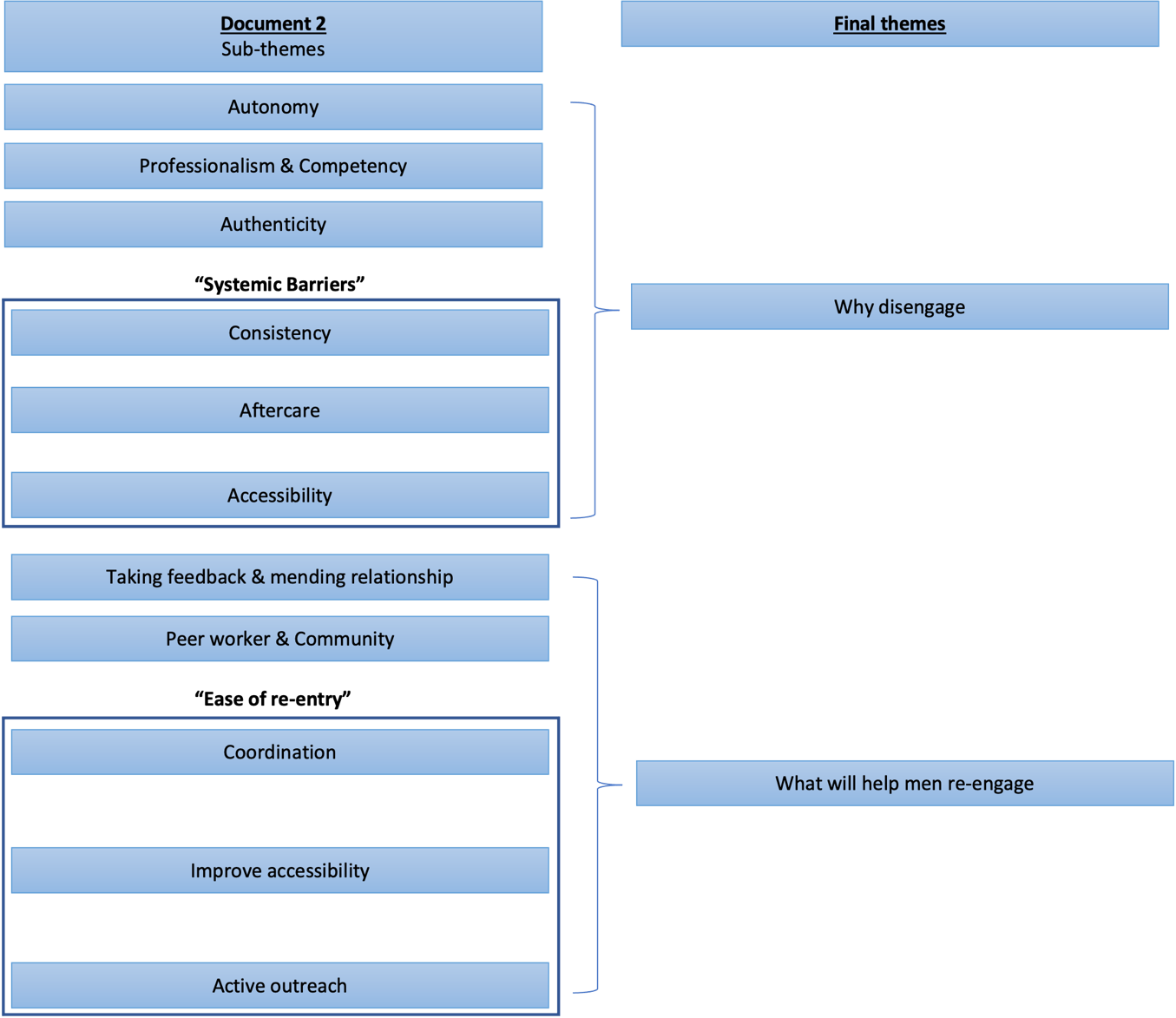

Supplement: sj-docx-1-jmh-10.1177_15579883231157971 – Supplemental material for Understanding Men’s Engagement and Disengagement When Seeking Support for Mental Health [file sj-docx-1-jmh-10.1177_15579883231157971.docx]
